# The Natural Cytotoxicity Receptor 1 Contribution to Early Clearance of *Streptococcus pneumoniae* and to Natural Killer-Macrophage Cross Talk

**DOI:** 10.1371/journal.pone.0023472

**Published:** 2011-08-22

**Authors:** Shirin Elhaik-Goldman, Daniel Kafka, Rami Yossef, Uzi Hadad, Moshe Elkabets, Alexandra Vallon-Eberhard, Luai Hulihel, Steffen Jung, Hormas Ghadially, Alex Braiman, Ron N. Apte, Ofer Mandelboim, Ron Dagan, Yaffa Mizrachi-Nebenzahl, Angel Porgador

**Affiliations:** 1 The Shraga Segal Department of Microbiology and Immunology and the National Institute for Biotechnology in the Negev, Ben Gurion University of the Negev, Beer Sheva, Israel; 2 Pediatric Infectious Disease Unit, Soroka University Medical Center, Ben Gurion University of the Negev, Beer Sheva, Israel; 3 Department of Immunology, Weizmann Institute of Science, Rehovot, Israel; 4 The Lautenberg Center for General and Tumor Immunology, The Hebrew University Hadassah Medical School, Jerusalem, Israel; Duke University Medical Center, United States of America

## Abstract

Natural killer (NK) cells serve as a crucial first line of defense against tumors, viral and bacterial infections. We studied the involvement of a principal activating natural killer cell receptor, natural cytotoxicity receptor 1 (NCR1), in the innate immune response to *S. pneumoniae* infection. Our results demonstrate that the presence of the NCR1 receptor is imperative for the early clearance of *S. pneumoniae*. We tied the ends *in vivo* by showing that deficiency in NCR1 resulted in reduced lung NK cell activation and lung IFNγ production at the early stages of *S. pneumoniae* infection. NCR1 did not mediate direct recognition of *S. pneumoniae*. Therefore, we studied the involvement of lung macrophages and dendritic cells (DC) as the mediators of NK-expressed NCR1 involvement in response to *S. pneumoniae*. *In vitro*, wild type BM-derived macrophages and DC expressed ligands to NCR1 and co-incubation of *S. pneumoniae*-infected macrophages/DC with NCR1-deficient NK cells resulted in significantly lesser IFNγ levels compared to NCR1-expressing NK cells. *In vivo*, ablation of lung macrophages and DC was detrimental to the early clearance of *S. pneumoniae*. NCR1-expressing mice had more potent alveolar macrophages as compared to NCR1-deficient mice. This result correlated with the higher fraction of NCR1-ligand^high^ lung macrophages, in NCR1-expressing mice, that had better phagocytic activity compared to NCR1-ligand^dull^ macrophages. Overall, our results point to the essential contribution of NK-expressed NCR1 in early response to *S. pneumoniae* infection and to NCR1-mediated interaction of NK and *S. pneumoniae* infected-macrophages and -DC.

## Introduction

The gram positive bacterium *Streptococcus pneumoniae* belongs to the commensal flora of the human respiratory tract. However *S*. *pneumoniae* also causes clinical infections including pneumoniae, meningitis and sepsis [1]. Pneumococcus is the fifth leading cause of death worldwide [2]. Considering the spread of antibiotic resistance to the bacterium, it is crucial to understand the host response to pneumococcal infection in order to improve therapy.

The host immune response to pneumococcal lung disease has been characterized as an intense inflammatory reaction, initially involving resident alveolar and interstitial macrophages, followed by lung infiltrating neutrophils [2]. Yet, the contribution of immune components, other than phagocytic cells, has also been demonstrated to be important [3]–[6]. It has emerged that chemokines and pro-inflammatory cytokines such as tumor necrosis factor alpha (TNFα), interleukin (IL)-6 and IL-1β have a crucial role in defense against *S. pneumoniae.* These mediators recruit and activate inflammatory cells to the site of infection. Several studies, including our own, showed that depletion or genetic ablation of these cytokines resulted in impaired host defense [7]–[9]. Interferon gamma (IFNγ) is another critical immunomodulator in early host defense against a variety of infections. IFNγ is a key activator of macrophage killing activity and also recruits circulating neutrophils and lymphocytes to the sites of infection. The role of IFNγ in natural immunity to *S. pneumoniae* infection is not clear as reports are contradictive [10]–[12]. Natural killer (NK) cells are bone marrow derived lymphocytes that constitute a key frontline defense against a wide range of pathogens such as viruses, bacteria, intracellular parasites [13]–[15], as well as tumors [16]. NK cells are believed to release the prominent fraction of the IFNγ evident during Gram-positive infection [17] and they are recruited to the lung during pneumococcal pneumoniae within 6 hours of infection [18]. Although NK cells can kill target cells spontaneously without prior stimulation, a delicate balance between inhibitory and activating receptors tightly regulate their activation. Among these, natural cytotoxicity receptor-1 (NCR1, also named NKp46) is the only receptor reported so far to be expressed specifically on NK cells in all mammals tested, including humans [19] and mice [20]. NKp46 is a transmembrane type i glycoprotein containing two immunoglobulin domains and positively charged arginine residue in the transmembrane domain, which associates with the CD3ζ or the FcεRIγ signaling adaptor molecules [19], [21] . Several *in vitro* studies have demonstrated that NKp46 is important in the recognition and destruction of various tumors [16], [22] and virus infected cells [23], [24]. In addition, we have recently demonstrated a critical function for murine NCR1 (murine NKp46) in the *in-vivo* eradication of influenza virus [25]. There are contradicting reports about the role of NK cells and IFNγ in pneumococcal infections, thus their exact effect is not yet defined. Rubins *et al*. showed that IFNγ^-/-^ mice demonstrated increased mortality during *S. pneumoniae* lung infection, suggesting a protective role for IFNγ in host response to pneumococcal disease [10]. In contrast, Rijneveld *et al.* demonstrated that IFNγ does not serve a protective role during pneumococcal pneumonia. IFNγR^-/-^ mice and IFNγ^-/-^ mice had relatively increased resistance to *S*. *pneumoniae* infection, exhibiting significantly fewer pneumococci in their lungs in comparison to wild type (WT) mice [11]. NK cells were shown to be detrimental in pneumococcal *pneumonia* and sepsis in immunocompromised mice; depletion of NK cells in SCID mice resulted in significantly lower bacteremia and inflammatory cytokine production [26]. In this present study we employed C57BL/6 mice in which a gene encoding GFP was inserted into the *Ncr1* locus, thereby rendering the *Ncr1* gene nonfunctional. Using these mice we assessed the involvement of the NCR1 receptor in the activation of NK cells following *S. pneumoniae* infection, and the role of NCR1 in the reciprocal interaction between NK cells, DC and macrophages following *S*. *pneumoniae* infection. The involvement of NCR1 was shown to contribute to NK cell activation together with *S. pneumoniae* clearance at early stages following inoculation.

## Materials and Methods

### Mice

C57BL/6 mouse strain *Ncr1* ^gfp/gfp^, *Ncr1* ^+/gfp^ and *Ncr1* ^+/+^ wild type littermates were used. In these mice, as described previously [25], the gene encoding the NCR1 receptor (*Ncr1*) was replaced with a green fluorescent protein (GFP) reporter cassette. This study involved also the use of the C57BL/6 mouse strain CD11c:Diphtheria toxin (DTx) receptor (DTR) transgenic mice (B6.FVB-Tg (Itgax-DTR/GFP)57Lan/J; The Jackson Laboratory) that carry a human DTR transgene under the murine CD11c promoter [27]. All experiments were done in the animal facilities of Ben Gurion University according to guidelines of the ethical committee.

### Bacteria

The *S. pneumoniae* strain WU2 (capsular serotype 3) was used in this study [28]. This strain was grown to mid-late log phase as determined by OD in Todd-Hewitt broth supplemented by yeast extract. Aliquots of bacteria were harvested by centrifugation, re-suspended in sterile PBS containing 10% glycerol and stored at −70°C. Colony-forming units (CFU) counts were verified in each experiment on blood agar plates at 37°C under microaerobic conditions.

### Infection of mice with *S. pneumoniae*

For mice infection, aliquots of bacteria were thawed rapidly and samples of serial 10-fold dilutions were plated onto blood agar plates for determining bacterial concentration. Each mouse was anaesthetized with Terrel isoflurane (MINRAD, New York, USA) and inoculated intranasally with 5×10^7^, 1×10^8^ or 4×10^8^ bacteria (in 25 µl PBS). Survival was monitored daily until 8 days after inoculation.

### Determination of bacterial load in the nasopharynx and lungs

Mice were sacrificed by Terrel isoflurane inhalation at 3, 6 and 24 h after inoculation. Nasopharynx and lungs were removed and homogenized in 1 ml of sterile PBS using the Polytron PT-10 homogenizer (Kinematika, Lucerne, Switzerland). Samples (25 µl) in serial dilutions were then plated onto blood agar plates and grown overnight (18 h) at 37°C in anaerobic jars for CFU determination.

### Real time PCR

Total RNA was extracted from C57BL/6 mice lungs by TRI-Reagent (Invitrogen, Carlsbad, CA, USA) according to the manufacturer's instructions. Purified RNA was converted to cDNA utilizing Promega's First Strand Synthesis (Madison, WI, USA). 20–100 ng RNA was subsequently used as a template for each Real-Time-PCR reaction. All PCR reactions for cytokine-specific mRNA were preformed on ABI PRISM 7500 Real-Time PCR System (ABI Applied Biosystems, USA) using proprietary cytokine-specific primers from ABI Applied Biosystems, USA. Relative cytokine mRNA levels were determined by normalization of signal with that for GAPDH mRNA. In initial studies, fivefold dilutions of cDNA generated a linear signal curve over at least a 30-fold range of cDNA concentrations. mRNA induction in pulsed lung tissue was reported as-fold increases over uninfected lung mice. We thank Mr. Shahar Dotan (Ben Gurion University of the Negev, Beer-Sheva, Israel) for supplement of the primers.

### Depletion of CD11c^+^ cells following Intratracheal (i.t.) instillation of DTx

PBS (80 µl) containing DTx (100 ng/gr, List Biological Laboratories) was applied to the mouse tracheae. Mice were lightly anesthetized using isoflurane and placed vertically and their tongues were pulled out. Using a long nasal-tip, liquid was placed at the top of tracheae and was then actively aspirated by the mouse. Gasping of treated mice verified liquid application to the alveolar space.

### Cell isolations and generation of Bone Marrow Derived DCs/Macrophages

For bronchoalveolar lavage (BAL), the trachea was exposed to allow insertion of a catheter, through which the lung was filled and washed 10 times with 5 ml of PBS without Ca^2+^/Mg^2+^. Bone marrow (BM)-derived DC (BMDC) and bone marrow (BM)-derived macrophages (BMMQ) were generated from Bone marrow which obtained from C57BL/6 mice by flushing femoral cells with a 23-gauge needle with RPMI. Low-density mononuclear bone marrow cells were isolated using red blood cell lysing buffer (SIGMA) and centrifuged. Cells (5×10^6^) were cultured in 10 cm tissue dishes with the fresh complete Iscove's Modified Dulbecco's Medium (cIMDM) with recombinant GM-CSF (100 ng/ml) or 20% Lymphocyte conditioned medium (LCM) respectively. The medium was changed every 2–3 days and replaced with fresh medium supplemented with GM-CSF (100 ng/ml) or 20% LCM respectively. For experiments, the slightly adherent cells (DC) and/or the adherent cells (macrophages) were harvested on days 6 to 8 of culture.

For purification of NK cells, spleens were harvested and purified from RBC using the ACK lysing buffer (Quality Biological, Inc., Gaithersburg, MD). NK cells were then negatively selected by EasySep NK Selection Kit (StemCell Technologies, Inc., Vancouver, Canada). The percentage of NK cells in the isolated population was evaluated using PE-conjugated anti-CD3 mAb and APC-conjugated anti-CD56 or anti-NK1.1 mAb (eBioscience) by flow cytometry. Recombinant IL-2 (100 IU/ml) was added in order to obtain a polyclonal NK cell population.

### Antibodies and Flow cytometry

Flow cytometry analysis was employed for analysis of cell surface marker expression. Splenocytes, total lung cells, BM cells (1×10^6^ per well) or bronchoalveolar lavage fluid (BALF) cells (0.5×10^6^ per well) were plated in 96-well U-bottom plates. Cells were washed and non specific binding was blocked with anti-CD16/CD32 in 0.5% FCS/0.5% mouse serum/PBS for 15 min on ice. Cells were then stained for 25 min with the following specific mAbs: Biotin-conjugated-anti-mF4/80, FITC-anti-mCD45, PE-anti-mNK1.1, PE-anti-mCD69, PE-anti-mCD115, PE-anti-mCD3, PE-anti-mCD11b, PercpCy5.5-anti-mCD11c, Pacific Blue-anti-mCD11b, Alexa flour 647-anti-mCD107a, APC-anti-NK1.1, APC-anti-mCD3, APC-anti human Fc-IgG, APC-conjugated streptavidin, all purchased from eBioscience. For staining with fusion-Igs, cells or bacteria were incubated with 4 or 2 µg of the mNCR1-Ig, mNKG2D-Ig, LIR1-Ig or CD99-Ig fusion proteins for 2 h at 4°C, washed, and stained with APC-conjugated-F(ab')2 goat-anti-human-IgG-Fc (109-136-098, minimal cross-reaction to bovine, horse and mouse serum proteins, Jackson Immuno Research, West Grove, PA). Staining and washing buffer consisted of 0.5% (w/v) BSA and 0.05% sodium azide in PBS. Propidium iodide or AmCyan dye was added prior to reading for exclusion of dead cells. Stained cells were analyzed using either FACSCalibur or FACSCanto II (Becton Dickinson, Mountain View). The data were then analyzed either with BD CELLQuest™ 3.3 software or FlowJo software version 6.3.4 (Tree Star). Fluorescence data was acquired using logarithmic amplification and reported fluorescence intensity units represent conversion of channel values according to the logarithmic scale (range 10^0^ to 10^4^). Results are shown as the geometric mean fluorescence intensity (MFI) of the stained populations.

### NK/BMDC and NK/BMMQ co-cultures

NK cells and BMDC or BMMQ co-cultures were preformed in RPMI 1640 +10% FCS in 24 well plates. Live *S. pneumoniae* were added to BMDC/BMMQ cultures (10^6^ cells /well) for 1.5 h at noted multiplicity of infection (MOI) ratio. NK cells were added to BMDC (1∶10) or BMMQ (1∶4) cultures 5 hours after infection followed by bacterial washing with Tetramycin (30 µg/ml), Gentamycin (50 µg/ml), Penicillin (100 U/ml) and Streptomycin (100 µg/ml). After additional incubation for 24 h cells were collected and analyzed.

### ELISA for IFNγ in supernatant

The concentration of IFNγ secreted into the media of BMDC/BMMQ cultures was measured using optimized standard sandwich ELISA. Media samples were centrifuge at 1200 rpm for 10 min and supernatant was collected and stored in -70°C. Recombinant mouse IFNγ (mIFNγ) which was used as standard, as well as the capture mAb, biotinylated mAb which was used for detection, and Streptavidin-HRP were purchased from BD-PharMingen. The samples were tested in triplicates. The concentration of the cytokine was determined relative to a standard curve of recombinant IFNγ (ranging from 10,000 pg/ml to 125 pg/ml at 1∶2 serial dilutions).

### Depletion of NK cells following intraperitoneal administration of anti-asialo GM1

NK cells were depleted using polyclonal anti-asialo-GM1 rabbit antibody (Wako Bioproducts, Neuss, Germany). Mice were injected with 50 µl of anti asialo-GM1 in 200 µl of PBS or with equivalent volume of PBS 24 h prior to intranasal infection with *S. pneumoniae.* 27 hours post-infection the lung and spleen were collected and subjected to a CFU assay and to FACS staining in order to check the efficacy of treatment.

### CFDA staining of bacteria

Bacteria were grown to mid-late log phase as determined by OD in Todd-Hewitt broth supplemented by yeast extract. Aliquots of bacteria were harvested by centrifugation (13000 rpm), re-suspended in sterile PBS and washed twice. Bacteria (1 ml suspension, 1*10^6^ cell concentration) were then stained with carboxyfluorescein diacetate (CFDA) using 200 µl of stock solution (0.5 mM), incubated with shaking for 30 min in 37°C, and washed twice following incubation.

### Immunocytochemistry

BALF cells were grown on chamber µ-slide (Ibidi, Germany), incubated alone or with 1 MOI CFDA-labeled *S. pneumonia* for 1 hour. Cells were then rinsed with PBS, fixed for 10 minutes in 2% paraformaldehyde and blocked with PB/A blocking solution (PBSX1, 5% BSA, 0.5%NaN3) for 30 minutes. Fixed cell were stained with mNCR1-Ig and rat anti mouse F4/80-biotin conjugated. Secondary staining was made using APC-conjugated-F(ab')2 goat-anti-human-IgG-Fc and PE-Cy7-Strepavidin. Nuclear staining was made using Hoechst 33342 1∶10000 (Fluka, Switzerland). All incubations were made in room temperature. All antibodies were diluted in PB/A. After each step the wells were rinsed for three times. Images acquisition and analysis were made using either Olympus IX70 fluroscentic microscope or Olympus Fluoview 1000 Laser Scanning Microscopy system equipped with a 40x/1.3 oil immersion objective.

### Statistical analysis

Results obtained from groups of 3 to 12 mice were expressed as the mean ±SD. Mann-Withney U-test analysis was used. Differences in the number of bacterial loads in the lungs were analyzed by using two-tailed Student's *t* test. Differences in cells number between two groups were analyzed by using two-tailed Student's t test or by one-way ANOVA. Values for p<0.05 were considered to be statistically significant.

## Results

### Survival of *Ncr1* ^gfp/gfp^, *Ncr1* ^+/gfp^ and *Ncr1* ^+/+^ mice following intranasal challenge with *S. pneumoniae*

**Figure 1:**
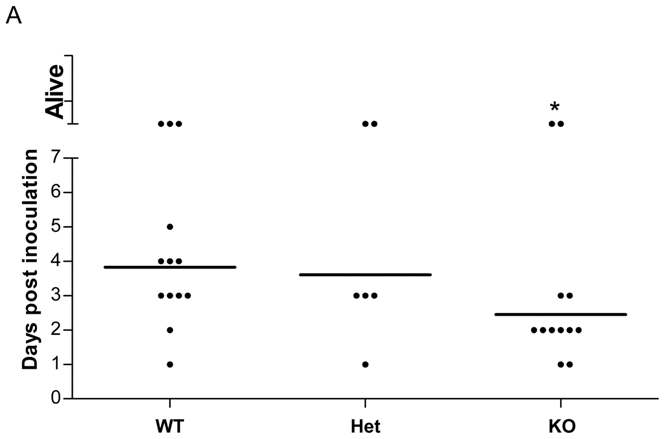
Survival of mice after intranasal inoculation with *S. pneumoniae*. (A) *Ncr1* ^+/+^ (WT, n = 13), *Ncr1* ^+/gfp^ (Het, n = 6) and *Ncr1* ^gfp/gfp^ (KO, n = 12) C57BL/6 mice were challenged intranasally with high lethal dose of *S. pneumoniae* strain WU2 in 25 µl PBS. Survival was monitored daily for 8 days. Results are the summary of two independent experiments. Statistical analysis revealed significant differences in survival rates between *Ncr1* ^+/+^ and *Ncr1* ^gfp/gfp^ mice, while *Ncr1* ^+/+^ mice had higher survivel rate (*p<0.05, two-tailed Mann-Withney U-test, line is for geometric mean).

We first aimed to evaluate the influence of deficiency of the activating natural killer cell receptor gene, *Ncr1*, on the survival of mice after infection with *S. pneumoniae*. C57BL/6 *Ncr1* ^gfp/gfp^, *Ncr1* ^+/gfp^ and *Ncr1* ^+/+^ WT littermate mice were infected with high lethal dose (4×10^8^) of *S. pneumoniae* serotype 3 strain WU2 and survival was monitored for 8 days (Fig. 1). The survival rate of *Ncr1* ^gfp/gfp^ was significantly lower than *Ncr1* ^+/+^ mice (p<0.05). The *Ncr1* ^+/gfp^ shows the same pattern of survival as *Ncr1* ^+/+^ but is not significantly different from the survival rate of *Ncr1* ^gfp/gfp^ mice due to small number of mice. Challenging the mice with 1×10^8^ or with 5×10^7^ of *S. pneumoniae* resulted in similar survival rate for all 3 mice groups (data not shown). These data suggest that the NCR1 receptor plays an important role in the survival of mice only following challenge with high dose of *S. pneumoniae*.

### Bacterial load in lungs of *Ncr1* ^gfp/gfp^, *Ncr1* ^+/gfp^ and *Ncr1* ^+/+^ mice following intranasal challenge with *S. pneumoniae*

**Figure 2:**
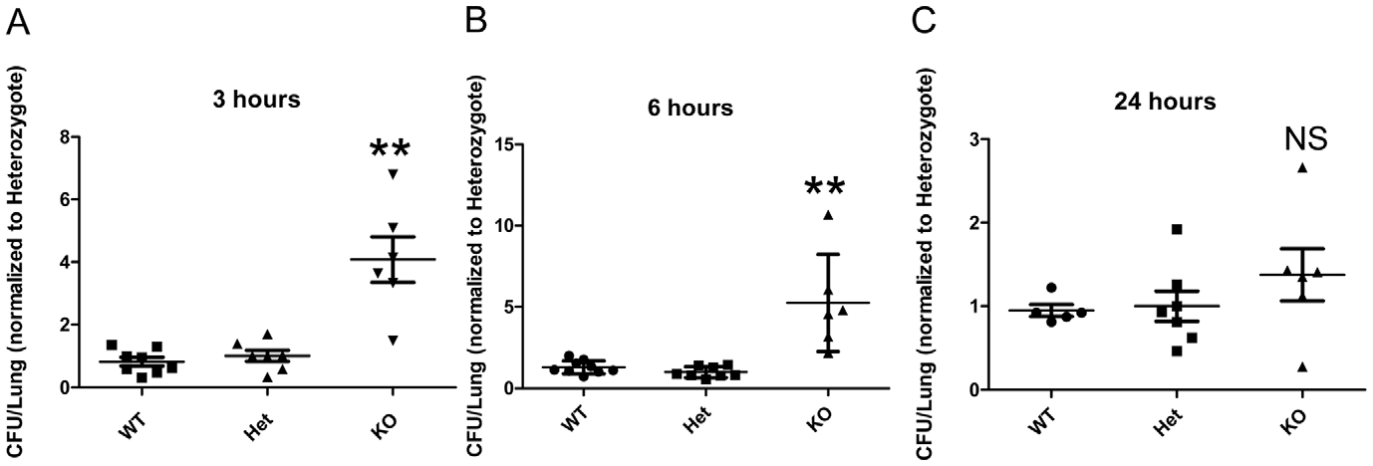
Bacterial load in lungs. *Ncr1* ^+/+^ (WT), *Ncr1* ^+/gfp^ (Het) and *Ncr1* ^gfp/gfp^ (KO) C57BL/6 mice were challenged intranasally with 5×10^7^ concentration of *S. pneumoniae* strain WU2. Lungs were harvested for CFU assessment: (A) 3 h after inoculation (n = 6 to 8), (B) 6 h (n = 5 to 9), and (C) 24 h (n = 5 to 6). Results are the summary of 3 independent experiments and were normalized according to the CFU of the Het group that its CFU average was considered as 1 in each experiment. ** p<0.01, compared with WT or with Het groups ±SD (two-tailed student t-test). NS, not significant compared with WT or with Het groups. Results are presented as vertical scatter plot of normalized CFU.

We next assessed the importance of the NCR1 receptor in the clearance of *S. pneumoniae* from the lungs at the early stages of infection. We compared the bacterial load in this organ of *Ncr1* ^gfp/gfp^, *Ncr1* ^+/gfp^ and *Ncr1* ^+/+^ mice, at 3, 6 and 24h after intranasal inoculation with 5×10^7^ *S. pneumoniae* strain WU2 (Fig. 2). In accordance with the survival results, 24 h after inoculation, the bacterial load in the lungs of *Ncr1* ^gfp/gfp^ mice was comparable to the levels observed in the lungs of *Ncr1* ^+/+^ and *Ncr1* ^+/gfp^ mice (Fig. 2C). In sharp contrast, during the first 6 hours of infection, the bacterial load in the lungs of *Ncr1* ^gfp/gfp^ mice was significantly higher than in *Ncr1* ^+/+^ and *Ncr1* ^+/gfp^ mice (Fig. 2A, 2B, p<0.01). The number of bacteria in the lungs of *Ncr1* ^+/+^ and *Ncr1* ^+/gfp^ mice was similar during all the above-described time points (Fig. 2A–C). This is in accordance with the observation that NK cells from *Ncr1* ^+/gfp^ mice are fully competent [25]. Overall, bacterial load numbers in the lungs of *Ncr1* ^+/+^ and *Ncr1* ^+/gfp^ mice increased 5.7 fold in average between 3 to 24 h post challenge, while for *Ncr1* ^gfp/gfp^ mice the average fold increase was only 1.7. These data suggest that the NCR1 receptor plays an important role in mediating the clearance of *S. pneumoniae* in the lungs early after infection.

### Contribution of NCR1 to NK cells activation by *S. pneumoniae*

**Figure 3:**
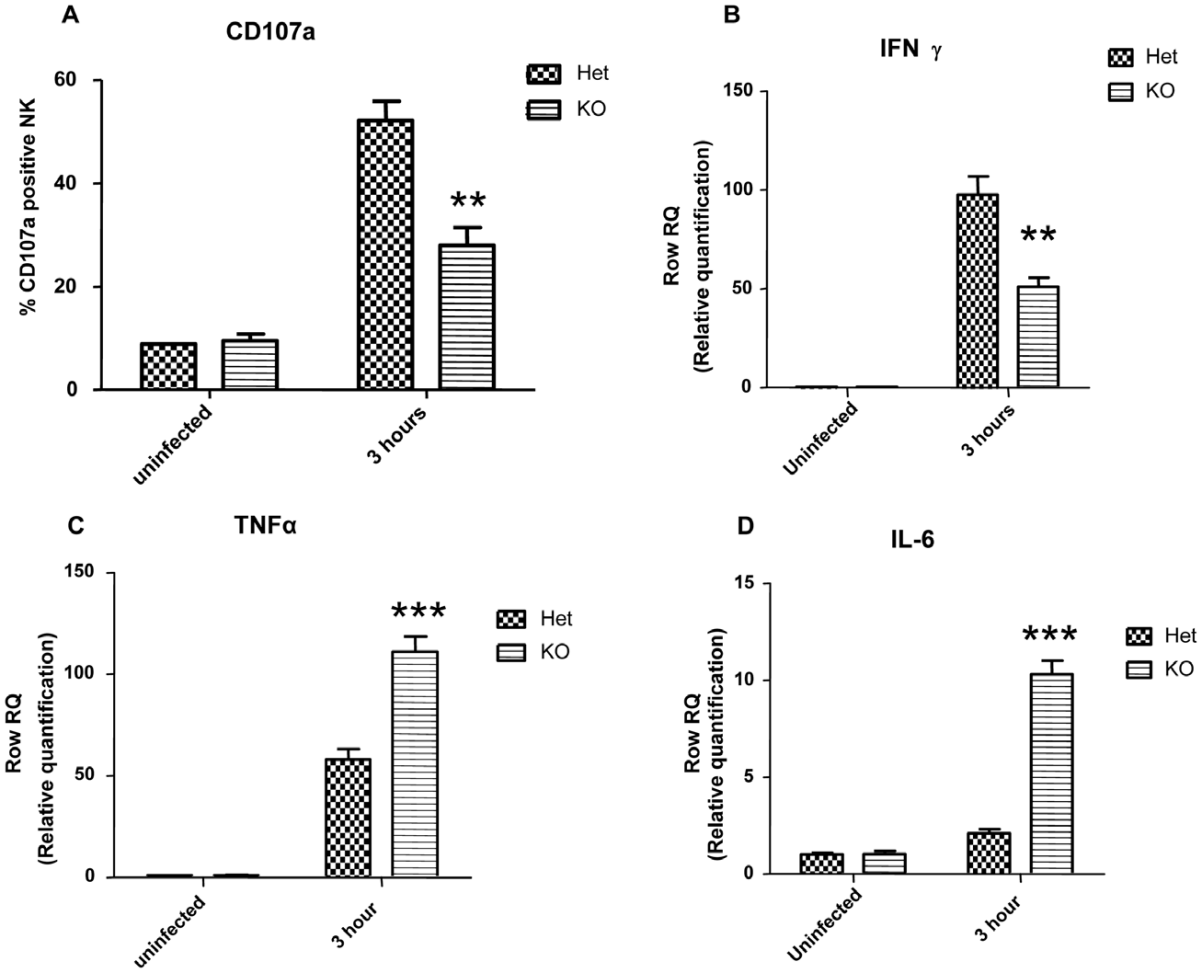
NK cells activation and cytokines mRNA levels in lungs following intranasal challenge with *S. pneumoniae*. Lungs were harvested from *Ncr1* ^+/gfp^ (Het group) and *Ncr1* ^gfp/gfp^ (KO group) C57BL/6 mice 3h after intranasal inoculation with 5×10^7^ CFU of *S. pneumoniae* strain WU2, and from non infected mice. (A) NK cells were gated as CD45^+^NK1.1^+^ and analyzed for the expression of membrane-associated CD107a by flow cytometry. The bar graphs show the fraction of CD107a positive NK cells (average of 3 mice per group, ±SD). ** p<0.01 compared to Het group in 3h-infected mice (ANOVA test). Total RNA was extracted from naïve lungs and from 3h-infected lungs of *Ncr1* ^+/gfp^ (Het) and *Ncr1* ^gfp/gfp^ (KO). Infection was performed with 5×10^7^ CFU of *S*. *pneumoniae.* Level of cytokines mRNA of IFNγ (n = 4), TNFα (n = 4) and IL-6 (n = 3) were analyzed by RT-PCR and calibrated to mRNA level of GAPDH. Results are from one representative experiment of two. *** p<0.001, ** p<0.01, compared with the Het group +SD (ANOVA test).

To further investigate the NCR1-dependent activation of NK cells following *S. pneumoniae* infection, we studied activation of NK cells and IFNγ production in the lungs 3 h following intranasal challenge with the bacteria. Enhancement of CD107a expression on NK cell membranes represents the fusion of secretory granules with the plasma membrane and not *de-novo* protein synthesis. Therefore, CD107a enhancement is a marker for NK cell activation at early stages reflecting cytokine secretion and release of the content of lytic granules [29]. *In vivo*, 3 h following *S. pneumoniae* challenge, membrane-associated expression of CD107a by lung NK cells appeared in both *Ncr1* ^+/gfp^ and *Ncr1* ^gfp/gfp^ mice compared to uninfected mice (p<0.05 for both, Fig. 3A). Yet, this increase was significantly higher in lung NK cells from NCR1-expressing mice (*Ncr1* ^+/gfp^) compared to NCR1-deficient mice (*Ncr1* ^gfp/gfp^) (52% vs. 28%, p<0.01, Fig. 3A). NK cell number in lungs of infected mice didn't differ between *Ncr1* ^+/gfp^ and *Ncr1* ^gfp/gfp^ (data not shown).

To assay local IFNγ production *in vivo*, total RNA was extracted from lungs of naïve and 3 h-infected *Ncr1* ^+/gfp^ and *Ncr1* ^gfp/gfp^ mice. IFNγ mRNA was quantified by real-time PCR. In accordance with the CD107a levels (Fig. 3B), IFNγ transcript in 3 h-infected lungs increased considerably compared to non-infected lungs for both *Ncr1* ^+/gfp^ and *Ncr1* ^gfp/gfp^ mice (Fig. 3B). Yet, the fold increment of IFNγ was significantly higher in NCR1-expressing mice compared to NCR1-deficient mice (Fig. 3B, p<0.01). Challenge with *S. pneumoniae* induces a local production of proinflammatory cytokines [2], [26], [30]. Therefore we investigated whether the observed differences in 3 h following challenge bacterial load could correlate with differential induction of other proinflammatory cytokines. TNFα and IL-6 mRNA levels were quantified. Similarly to the IFNγ, TNFα and IL-6 transcripts in the lung were greatly induced 3 h following challenge (Fig. 3C, D). However, the opposite trend to IFNγ results was observed when comparing between NCR1-expressing and -deficient mice; fold increase was significantly higher in *Ncr1* ^gfp/gfp^ mice (Fig. 3C, D, p<0.001). This data suggest that the NCR1 receptor plays an important role in activation of NK cells during early stages of *S. pneumoniae* infection by activating IFNγ production and enhancing CD107a expression.

### Bacterial load in the lungs of NK-depleted mice following intranasal challenge with *S. pneumoniae*

**Figure 4:**
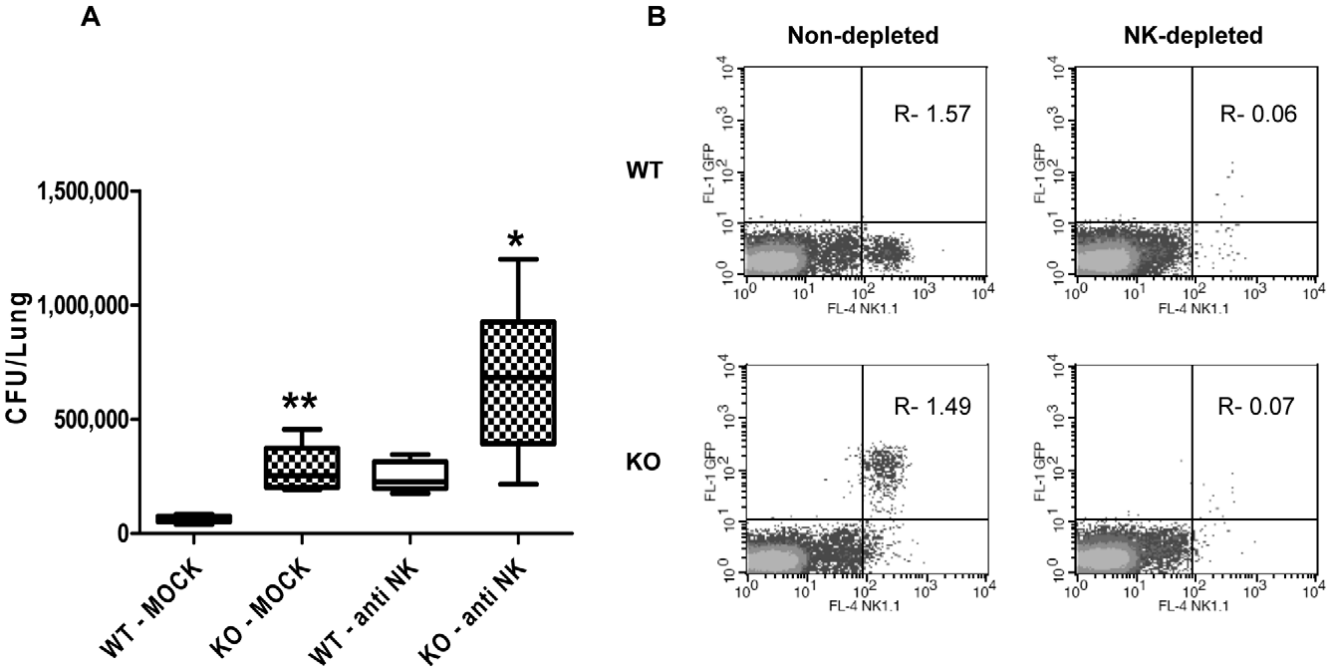
Bacterial load in lungs following *in vivo* depletion of NK cells. *Ncr1* ^+/+^ and *Ncr1* ^gfp/gfp^ C57BL/6 mice treated intraperitoneally either with 50 µl of anti asialo GM1 (WT-anti NK and KO-anti NK) or with 50 µl of PBS (mock treatment, WT-mock and KO-mock). 24 h after anti asialo GM1 or mock treatment, all mice were challenged with 5×10^7^ CFU of *S. pneumoniae* strain WU2 and lungs were harvested 3 h after challenge. (A) CFU of *S. pneumoniae* in lungs, 3 h after bacterial challenge (n = 3 to 5 per group). Results are presented as box plot of CFU. ** p<0.01 compared with WT-mock; * p<0.05, compared with WT-anti NK, ± SD (ANOVA test). (B) Flow cytometry analysis of spleen cells from depleted and non-depleted mice for CD3 negative-gated cell population showing NK1.1 versus GFP expression. Analysis was made 24 h after depletion and 3 h following bacterial challenge (representative mouse per each panel). Numbers indicate percentage of gated cells (LR+UR) from CD3^−^ cells. Results are from one representative experiment of two.

To validate the role of NK cells in mediating early clearance of *S. pneumoniae* in the lungs, we depleted NK cells using anti-asialo GM1 (one injection of 50 µl per mouse). More than 95% of NK cells were removed following depletion as tested 24 h later (Fig. 4B, shown for both *Ncr1* ^gfp/gfp^ and *Ncr1* ^+/+^ groups). Twenty-four hours following NK cell depletion or mock treatment, we challenged *Ncr1* ^gfp/gfp^ and *Ncr1* ^+/+^ mice with 5×10^7^ CFU *S. pneumoniae* strain WU2 and compared the bacterial load in the lungs 3 h later (Fig. 4A). In both mice groups, depletion of NK cells 24 h prior to infection resulted in a significant increase of bacterial load 3 h following inoculation compared to the mock-treated mice (3.8 and 2.57 fold increase, p<0.01 and p<0.05 for *Ncr1* ^+/+^ and *Ncr1* ^gfp/gfp^, respectively). These results point to the involvement of NK cells in early clearance of *S. pneumoniae* challenge. As expected (Fig. 4A), 3 h after inoculation, bacterial load in mock-treated *Ncr1* ^gfp/gfp^ was significantly higher compared to mock-treated *Ncr1* ^+/+^ mice (4.4 fold increase, p<0.01). Increased bacterial load was also observed between NK-depleted *Ncr1* ^gfp/gfp^ and NK-depleted *Ncr1* ^+/+^ mice, yet to a lower extent (2.9 fold, p<0.05). This indicates that in addition to NK cells contribution to early clearance upon challenge, other cells in *Ncr1* ^+/+^ mice, e.g. phagocytes, might be better conditioned to cope with *S. pneumoniae* infection at early stages.

### Bacterial load in the macrophage/ DC-depleted lungs following intranasal challenge with *S. pneumoniae*

**Figure 5:**
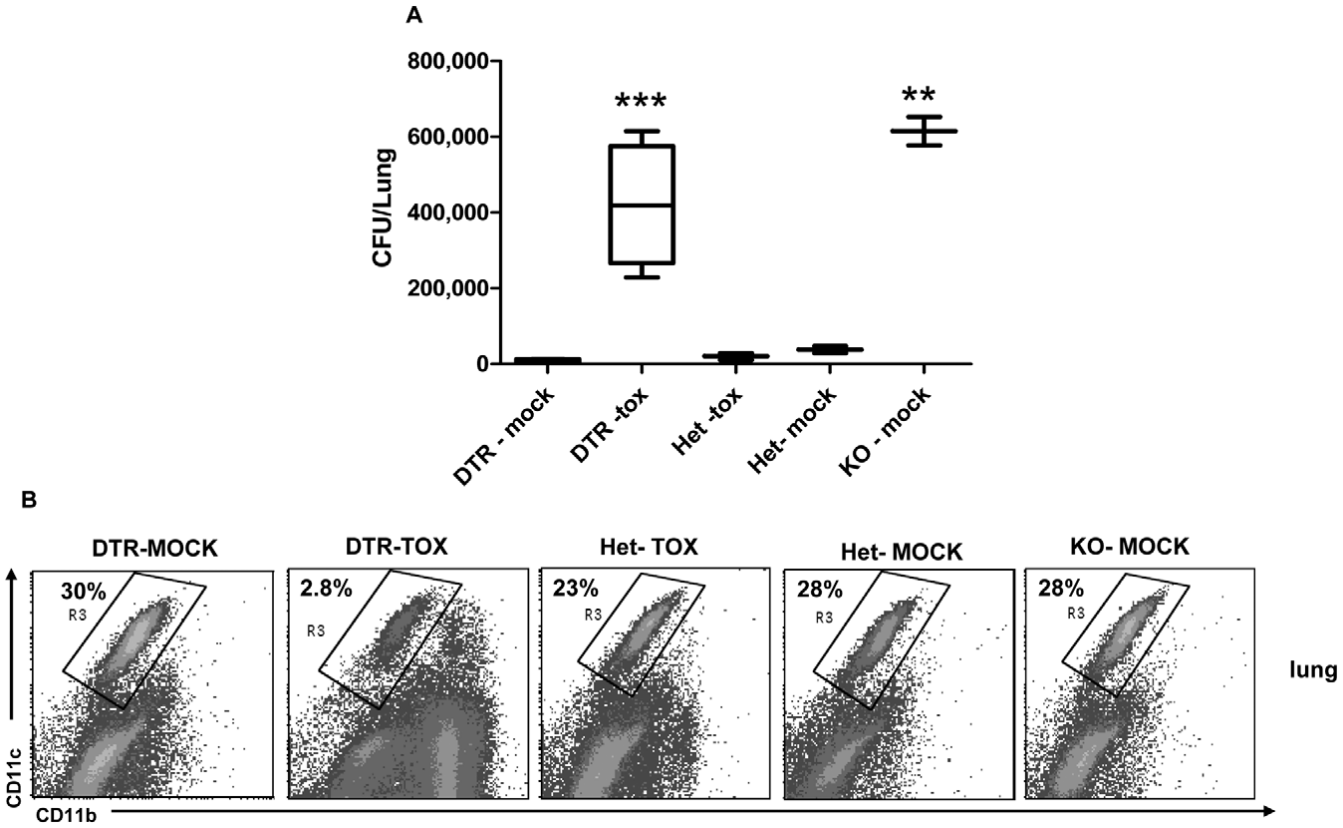
Bacterial load in lungs following *in vivo* ablation of lung CD11c^+^ cells. CD11c:DTR mice (C57BL/6 genetic background) were treated intratracheally with DTx (100 ng/gr, DTR-tox group). CD11c:DTR littermates treated intratracheally with PBS (‘DTR-mock’) served as control. *Ncr1* ^+/gfp^ C57BL/6 mice treated intratracheally with DTx (‘Het-tox’) or with PBS (‘Het-mock’) and *Ncr1* ^gfp/gfp^ C57BL/6 mice treated with PBS (‘KO-mock’) served as additional control groups. One day after DTx or mock treatment, all mice were challenged with 5×10^7^ CFU of *S*. *pneumoniae* strain WU2 and lungs were harvested 3 h after challenge. (A) CFU of *S*. *pneumoniae* in lungs, 3 h after bacterial challenge (n = 3 to 5 per group). Results are presented as box plot of CFU . *** p<0.001 compared with DTR-mock; ** p<0.01, compared with Het-mock, ± SD (ANOVA test). (B) Flow cytometry analysis of lung cells for CD11c and CD11b expression, 3 h after bacterial challenge (representative mouse). Numbers indicate percentage of gated cells from total white blood cells. Results are from one representative experiment of two.

To assay *in vivo* the contribution of lung phagocytes to *S. pneumoniae* clearance at early stages following infection, the extent of bacterial load in *Ncr1* ^gfp/gfp^, *Ncr1* ^+/gfp^ and mice ablated from lung CD11c^+^ mononuclear phagocytes was compared. We took advantage of CD11c:DTR transgenic mice that allow a specific depletion of CD11c^high^ cells [27]. The intratracheal DTx installation into CD11c:DTR transgenic mice results in the specific local ablation of CD11c^+^ lung mononuclear phagocytes, including macrophages and DC [27] (Fig. 5B). Mice were inoculated with 5×10^7^ *S. pneumoniae* serotype 3 strain WU2, and we assessed the number of bacteria in the lungs DTx- and mock-treated CD11c: DTR mice, *Ncr1* ^+/gfp^ and *Ncr1* ^gfp/gfp^ mice 3 h after inoculation (Fig. 5A). The bacterial load in the lungs of DTx-treated CD11c: DTR mice was significantly higher than the load in the lungs of mock-treated CD11c: DTR mice (p<0.001), and was similar to that of mock-treated *Ncr1* ^gfp/gfp^ mice. DTx-treated and mock-treated *Ncr1* ^+/gfp^ mice had low bacterial load similar to that of mock-treated CD11c:DTR mice. Fold increase between mock-treated and DTx-treated CD11c: DTR mice was similar to that between mock-treated *Ncr1* ^+/gfp^ and *Ncr1* ^gfp/gfp^ (6 fold increase, p<0.01). Thus, lung CD11c^+^ mononuclear phagocytes play a major role in the clearance of *S. pneumoniae* inoculated intranasally.

### NCR1 involvement in NK cells activation following infection of bone marrow-derived macrophages and bone marrow-derived DC with *S. pneumoniae*

**Figure 6:**
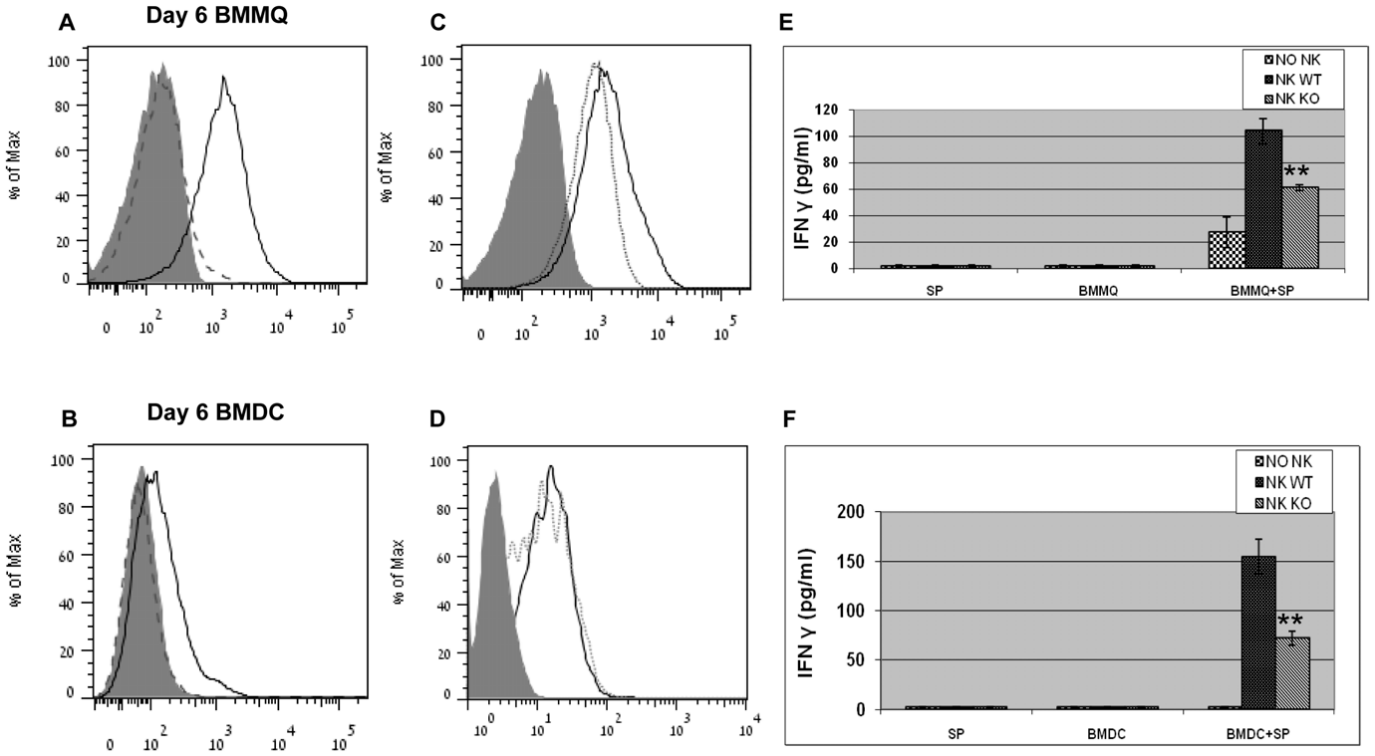
NCR1 involvement in NK cells activation following infection of BMMQ/ BMDC with *S. pneumoniae.* (A-D) Six-day culture of BMMQ and BMDC grown from *Ncr1* ^+/+^ BM cells (C57BL/6) were stained with mNCR1-Ig, mNKG2D-Ig and LIR1-Ig fusion proteins. Shown are representative overlays of F4/80^+^CD115^+^ BMMQ (A, C) and CD11c^+^ BMDC (B, D) demonstrating staining with mNCR1-Ig (solid line, A-D), mNKG2D-Ig (dotted line, C–D), LIR1-Ig (dashed line, A–B) and with goat anti-human IgG (grey-filled, A–D). Shown is one representative experiment out of four to five independent experiments. In some experiments, NKG2D-Ig staining was higher compared to NCR1-Ig staining. (E–F) Six-day culture- BMMQ (E) and -BMDC (F) grown from *Ncr1* ^+/+^ BM cells (C57BL/6) were infected with 1 MOI of *S. pneumoniae* for 1.5 h, then bacteria were removed and antibiotics were added. In parallel, NK cells were isolated from splenocytes of *Ncr1* ^+/+^ (‘NK WT’) and *Ncr1* ^gfp/gfp^ (‘NK KO’) C57BL/6 mice. Isolated NK were incubated with uninfected BMMQ or BMDC, or with *S. pneumoniae*-infected BMMQ or BMDC (‘BMMQ+SP’, ‘BMDC+SP’) after removal of bacteria for additional 18 hours. NK-BMMQ ratio was 1∶4 and NK-BMDC ration was 1∶10. IFNγ concentration levels in the co-culture supernatants were determined by ELISA. Results are from one representative experiment out of two. ** p<0.01 compared with IFNγ levels of NK WT incubated with infected BMMQ/BMDC, ± SD (ANOVA test).

We studied the expression of ligands to NCR1 by BMMQ and BMDC. We stained BMMQ and BMDC from 6-day cultures with mNCR1-Ig and mNKG2D-Ig fusion proteins. Human protein LIR1-Ig served as negative control for the staining. F4/80^+^CD115^+^ BMMQ and CD11c^+^ BMDC were stained positively with the mNCR1-Ig (Fig. 6A–D) and mNKG2D-Ig (Fig. 6C–D). The positive staining was specific, as no staining was observed with the control fusion protein LIR1-Ig (Fig. 6A–B). These results imply that macrophages and DC are able to express ligands to NCR1 and could be involved in the innate immune response of NCR1^+/+^ mice to *S. pneumoniae* challenge. We then investigated whether *S. pneumoniae*-infected BMMQ or BMDC could activate NK cells via the NCR1 receptor; we studied IFNγ levels in the co-cultures of BMMQ/BMDC from WT mice with NK cells from NCR1-expressing and NCR1-deficient isolated NK cells. In brief, WT BMMQ/BMDC from 6-day culture were infected with *S*. *pneumoniae* for 1.5 hr, then bacteria were washed and antibiotics were added; Infected BMMQ/BMDC were then co-cultured with NK cells from either *Ncr1* ^+/+^ or *Ncr1* ^gfp/gfp^ C57BL/6 mice for 18 hrs and IFNγ levels in the supernatant were assayed.

IFNγ levels were zero to negligible for NK cell cultures without BMMQ/BMDC even in the presence of *S*. *pneumoniae* (Fig. 6E–F, ‘SP’). Similarly, no IFNγ was detected when NK cells were co-cultured with non-infected BMMQ/BMDC (Fig. 6E–F, ‘BMMQ’, ‘BMDC’). Co-cultures of *Ncr1* ^gfp/gfp^-derived NK cells with WT BMMQ/BMDC contained IFNγ, yet significantly less than IFNγ levels in co-cultures of *Ncr1* ^+/+^-derived NK cells with WT BMMQ/ BMDC (p<0.01, Fig. 6E–F ‘BMMQ+SP’, ‘BMDC+SP’). To summarize, these results indicate a significance of NCR1 for NK cell activation following the cross-talk with *S. pneumoniae*-infected BMMQ and BMDC.

### NCR1 ligand expression on macrophages and DC

**Figure 7:**
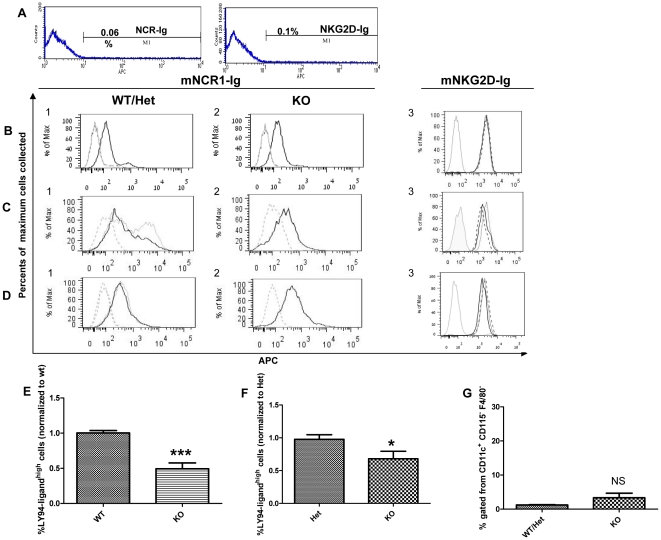
NCR1 ligand expression on *S*. *pneumoniae* and naïve macrophages and DC. (A) Fixed bacteria were stained with mNCR1-Ig (left panel) and mNKG2D-Ig (right panel) fusion proteins. Shown are representative overlays of demonstrating staining with the fusion protein (bold line) and with goat anti-human IgG (plain line). Both lines overlapped. Cells from lung lavage (B, E, F), BM (C, E, F, G) and spleen (D) of C57BL/6 *Ncr1* ^+/+^ (WT), *Ncr1* ^+/gfp^ (Het) and *Ncr1* ^gfp/gfp^ (KO) naive mice were stained with mNCR1-Ig, mNKG2D-Ig, LIR1-Ig (B) or CD99-Ig (C, D) and with macrophages/DC markers (F4/80, CD115, CD11c). Panels B-F were gated on macrophages (F4/80^+^CD115^+^) and panel G was gated on DC (CD11c^+^). Panels B-D show overlays of mNCR1-Ig staining as follows: bold line for *Ncr1* ^+/+^ (B1, C1, D1) or *Ncr1* ^gfp/gfp^ (B2, C2, D2), hairline for *Ncr1* ^+/gfp^ (C1, D1); LIR1-Ig staining (grey line for B1, B2); CD99-Ig staining (dashed line for C1, C2, D1, D2); and goat anti-human IgG (grey line for B3, C3, D3). mNKG2D-Ig staining for all mice types is shown is the right panel (B3, C3 and D3: *Ncr1* ^+/+^ in bold line, *Ncr1* ^+/gfp^ in dashed line and *Ncr1* ^gfp/gfp^ in dotted line). The bar graphs (E–G) show the fraction of NCR1-ligand^high^ BAL and BM-gated macrophages for *Ncr1* ^+/+^ vs. *Ncr1* ^gfp/gfp^ (E), the fraction of NCR1-ligand^high^ BAL and BM-gated macrophages for *Ncr1* ^+/gfp^ vs. *Ncr1* ^gfp/gfp^ (F) and NCR1-ligand^high^ BM-gated DC (G). Results in E are normalized to *Ncr1* ^+/+^ (n = 12 and 16 mice per group). Results in F are normalized to *Ncr1* ^+/gfp^ (n = 12 and 15 mice per group). *** p<0.001 and * p<0.05 compared to WT/Het group +SD (two-tailed student t-test). NS, non significant compared to WT/Het group. Results (B–D) are from one representative experiment out of six to seven.

To further investigate NK cell-expressed NCR1 involvement in *S*. *pneumoniae,* we first tested whether NCR1 ligands are expressed by the bacteria. A recent study suggested that NKp44 on human NK cells may be involved in the direct recognition of bacterial pathogens [31]. To investigate whether NCR1 could interact directly with the bacterium, we stained *S*. *pneumoniae* with soluble mNCR1-Ig and mNKG2D-Ig fusion proteins. Both proteins did not stain *S*. *pneumoniae* indicating that neither NKG2D- nor NCR1-ligands are expressed on the bacteria (Fig. 7A). These results imply that the involvement of NCR1 in *S. pneumoniae* infection could be indirectly mediated via the interaction of NK cells with NCR1 ligand-expressing macrophages or DC. The importance of the lung macrophage response during infection with *S*. *pneumoniae* implied from Fig. 5, and knowing that macrophages comprise 98% of lung lavage (BAL) leukocytes cells, led us to investigate the phenotypic characterization of macrophages in lung BAL and whether the presence of the NCR1 receptor in mice plays a role in this phenotype. Cells from lung BAL, BM and spleen were harvested from *Ncr1* ^+/+^, *Ncr1* ^+/gfp^ and *Ncr1* ^gfp/gfp^ naïve C57BL/6 mice and stained with mNCR1-Ig and mNKG2D-Ig for detection of their ligands. Human fusion proteins LIR1-Ig and CD99-Ig served as negative staining controls. Staining with mNCR1-Ig of lung BAL showed two different sub-populations: F4/80^+^CD115^+^NCR1-ligand^high^ and F4/80^+^CD115^+^NCR1-ligand^dull^ (Fig. 7B). Similarly, staining of BM F4/80^+^CD115^+^ macrophages also revealed the NCR1-ligand^high^ and NCR1-ligand^dull^ sub-populations (Fig. 7C). The NCR1-ligand^high^ macrophage sub-population was significantly higher in BAL and BM of *Ncr1* ^+/+^ compared to *Ncr1* ^gfp/gfp^ (2 fold, P<0.001, Fig. 7E); likewise, it was higher in lung and BM of *Ncr1* ^+/gfp^ compared to *Ncr1* ^gfp/gfp^ (1.4 fold, P<0.05, Fig. 7F). The results in panels 7E-F represent normalized values as the actual fraction of NCR1-ligand^high^ macrophage sub-population varied between experiments and mice and ranged between 5% to 25% for *Ncr1* ^+/+^ mice. Yet, it was significantly lower in lung BAL and BM of *Ncr1* ^gfp/gfp^ mice (Fig. 7E–F). Notably, the NCR1-ligand^high^ BAL macrophages had higher expression of CD115 and residual staining with LIR1-Ig that might point to high expression of Fc receptors though the blocking of those receptors during staining (see Methods). The staining of macrophages from spleen did not reveal the NCR1-ligand^high^ sub-population in any of the three mice types (Fig. 7D). We observed this direct correlation between NCR1 presence and NCR1-ligand^high^ macrophage sub-population in additional genetic backgrounds of mice (129/Sv and BALB/c, data not shown). This phenomenon was not observed for the staining with mNKG2D-Ig. Macrophages from lung, BM and spleen did not manifest heterogeneous phenotype for NKG2D-ligands expression which did not differ between *Ncr1* ^+/+^, *Ncr1* ^+/gfp^ and *Ncr1* ^gfp/gfp^ (Fig. 7B, C and D). In addition, we studied CD11c^+^ DC in both BM and spleen. The NCR1-ligand^high^ DC sub-population in BM and spleen (data not shown) was low to negligible and did not differ significantly between the mice groups (1.4% vs. 3.3%, Fig. 7G). These results show, for the first time, a unique macrophage sub-population, located in BM and lung, which is characterized by high expression of ligands for NCR1.

### NCR1 contribution to activation of alveolar macrophages

**Figure 8:**
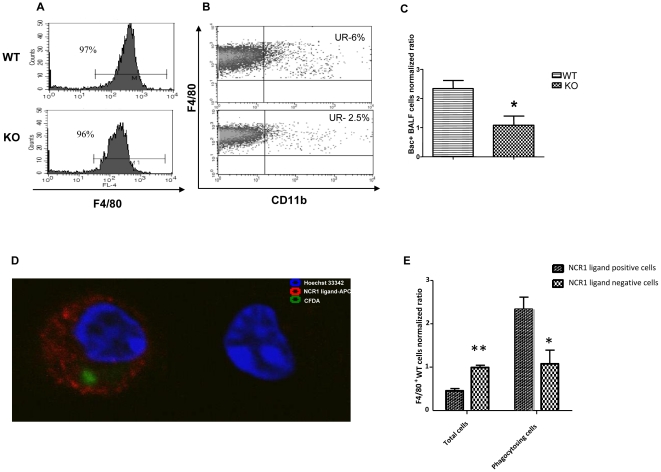
NCR1 contribution to activation of naïve alveolar macrophages. BALF cells were harvested from *Ncr1* ^+/+^ and *Ncr1* ^gfp/gfp^ naïve C57BL/6 lungs. Alveolar macrophages were gated by staining with mAbs to CD45 and F4/80 (A) and gated cells were analyzed for the expression of CD11b (B) by flow cytometry. Numbers indicate percentage of alveolar macrophages in BALF (A) and fraction of CD11b positive alveolar macrophages (B). Results are from one representative experiment of five. (C) BALF cells taken from *NCR1* ^+/+^ (WT) and *NCR1* ^gfp/gfp^ (KO) mice were incubated with 1 MOI CFDA-labeled *S. pneumonia* for 2 h. Cells were then washed and visualized with a fluorescent microscope. The bar graph (C) shows the average of fluorescent BALF cells (bac positive) from 11 microscopic fields. Numbers were normalized to the average of fluorescent BALF cells from *NCR1* ^gfp/gfp^ mice, which was considered as 1. *p<0.05 compared to *NCR1* ^+/+^ group, +SD (ANOVA test). Results are from one representative experiment out of two. (D–E) *Ncr1* ^+/+^ BALF were incubated with 1 MOI CFDA-labeled *S. pneumonia* for 1 hr. Following wash of bacteria, cells were fixed and stained with mNCR1-Ig and biotin-conjugated-rat anti mouse F4/80. Secondary staining was performed using APC-conjugated-F(ab')2 goat-anti-human-IgG-Fc and PE-Cy7-Strepavidin. Hoechst 33342 was employed for nuclear staining. Cells were washed and visualized with Confocal microscope. (D) Representative confocal image of NCR1-ligand^high^ (bac positive) and NCR1-ligand^none^ (bac negative) macrophages (F4/80 staining is not shown) (magnification X400). (E) We analyzed 52 random fields from 6 independent experiments and counted F4/80^+^ NCR1-ligand^high^ and NCR1-ligand^none^ cells, either positive or not for bacteria. The average number of NCR1-ligand^high^ and NCR1-ligand^none^ is shown normalized to NCR1-ligand^none^, which its average was considered as 1. (left two columns of the graph, **p<0.01 compared to NCR1-ligand^high^ group, +SD (two-tailed student t-test)). We then calculated the ratio of bacteria-positive for each of the two populations and normalized the results according to the fraction of NCR1-ligand^none&bac+^ of the total NCR1-ligand^none^ cells. Right two columns of graph shows the normalized fraction of bacteria positive cells for the two macrophage populations (NCR1-ligand^high^ and NCR1-ligand^none^). *p<0.05 compared to NCR1-ligand^high^ group, +SD (two-tailed student t-test).

We further investigated the NCR1 involvement in the activation of naïve alveolar macrophages. Macrophage activation state can be determined by (i) up regulation of activation molecules and by (ii) phagocytic capacity. BALF cells were taken from *Ncr1* ^+/+^ or *Ncr1* ^gfp/gfp^ C57BL/6 mice lungs and the alveolar macrophages were gated by staining with antibodies to CD45 and F4/80 and analyzed for the expression of CD11b activation markers. CD45^+^F4/80^+^ cells consisted the majority of BALF cells for both *Ncr1* ^+/+^ and *Ncr1* ^gfp/gfp^ mice types (97% and 96% respectively, Fig. 8A). Higher fraction of CD11b^+^ alveolar macrophages was observed in *Ncr1* ^+/+^ compared to *Ncr1* ^gfp/gfp^ mice (6% vs. 2.5%, Fig. 8B). Macrophages number in the BALF was similar between the *Ncr1* ^+/gfp^ and *Ncr1* ^gfp/gfp^ mice group as determined by cell counting (403,333 vs. 487,667 per lung; data not shown). Phagocytic capacity was evaluated by the incubation of CFDA-stained bacteria with freshly isolated BALF cells and direct visualization with either conventional fluorescent or confocal microscope. We observed that significantly higher numbers of BALF cells from *Ncr1* ^+/+^ mice were associated with CFDA-stained bacteria as compared to BALF cells from *Ncr1* ^gfp/gfp^ mice (2.2 fold, p<0.05, Fig. 8C). Confocal-based analysis of WT BALF cells showed that F4/80^+^ cells were either NCR1-ligand^high^ or NCR1-ligand^none^ (Fig. 8D, representative image). The ratio between the two populations (0.3 vs. 1, p<0.01, Fig. 8E) was similar to the results obtained by flow cytometry (Fig. 7E). The NCR1-ligand^dull^ observed by flow cytometry parallel to the NCR1-ligand^none^ observed by confocal microscopy. Importantly, there was a significantly higher proportion of phagocytosed CFDA-stained bacteria in F4/80^+^/NCR1-ligand^high^ as compared to F4/80^+^/NCR1-ligand^none^ (2.6 fold, p<0.05, Fig. 8E). These results imply that high NCR1 ligand expression is correlated with enhanced phagocytic activity of macrophages and further support our assumption that NCR1 expression by NK cells is associated with priming of alveolar macrophages.

## Discussion

NK cells constitute a key frontline defense against a range of viruses and bacteria. Different studies have demonstrated the importance of NK cells in controlling bacterial infections in mice, in particular *with Shigella flexneri* [32]. Human NKp46 is a key NK activating receptor that was reported to be involved in the response against *Mycobacterium tuberculosis* [14]. In the current study, we investigated the involvement of the mouse NCR1 (murine NKp46) in the innate immune response to *S. pneumoniae* infection. We showed the following: (i) NCR1 receptor plays a role in *S. pneumoniae* induced mortality in mice only when high challenge doses are applied (Fig. 1); yet NCR1 plays an imperative role in reducing *S. pneumoniae* bacterial load in the lungs at early stages after infection. (Fig. 2); (ii) *In vivo*, NCR1 expression activates IFNγ production in the lung and enhances the expression on lung NK of the membrane-associated CD107a early after infection with *S. pneumoniae* (Fig. 3); (iii) *In vivo*, NK cells, lung macrophages and/or DC are imperative for early clearance of *S. pneumoniae* (Fig. 4, 5); (iv) *In vitro*, NCR1 is important for NK cells activation during the interaction with *S. pneumoniae*-infected BMMQ and BMDC (Fig. 6); (v) NCR1 presence in mice is directly correlated with the magnitude of NCR1-ligand^high^ macrophage sub-population, located in lungs and BM (Fig. 7); and (vi) NCR1 presence in mice is correlated with higher activation state and phagocytic capacity of alveolar macrophages (Fig. 8). Taken together, these findings suggest that NCR1 plays an important role in mediating the killing of *S. pneumoniae* in the lungs during the initial stages of infection.

We studied survival rate following *S. pneumoniae* infection of *Ncr1* ^gfp/gfp^, *Ncr1* ^+/gfp^ and *Ncr1* ^+/+^ C57BL/6 mice. Deficiency of the key NK activating receptor, NCR1, resulted in lower survival rate of mice infected with high lethal dose. Lower lethal doses did not induce differences in survival rate; yet, during the first 6 hours of infection, a significantly reduced bacterial load was observed in the lungs of *Ncr1* ^+/+^ and *Ncr1* ^+/gfp^ mice compared to *Ncr1* ^gfp/gfp^ mice. In accordance with the survival results following lower lethal doses challenge, 24 h after inoculation, bacterial load in the lungs of *Ncr1* ^gfp/gfp^ mice was comparable to the levels observed in the lungs of *Ncr1* ^+/+^ and *Ncr1* ^+/gfp^ mice. These results suggest that the NCR1 receptor plays an important role in mediating the clearance of *S. pneumoniae* in the lungs only during the initial stages of infection. In the late stages of infection, the intense inflammatory response that evolves as a result of the large number of bacteria in the lungs may conceal the NCR1 receptor effect. Alternatively, since inflammation contribute to *S. pneumoniae* proliferation in lungs [1], [33] and NK cell activity and IFNγ could enhance inflammation [11], the lesser contribution of NCR1-deficient NK cells to inflammation could oppose the picture in the later stages of the infection. This is in accordance with the report on the detrimental effect of NK cells in *S. pneumoniae* infection [26]. The plausible divergent contribution of NCR1-mediated IFNγ production at early stages (clearance of *S. pneumoniae*) and late stages (enhancement of inflammation) could also explain the contradictory reports on the role of IFNγ in response to *S. pneumoniae* [10]-[12].

No differences in the bacterial load and survival between the *Ncr1* ^+/+^ and the *Ncr1* ^+/gfp^ mice could be observed. This was in accordance with a previous study, which demonstrated that NK cells from *Ncr1* ^+/gfp^ mice are fully functional while NK cells from *Ncr1* ^gfp/gfp^ mice manifest reduced activity following influenza infection and tumor development [25]. The reduced bacterial load in the *Ncr1^+/+^* and *Ncr1* ^+/gfp^ mice suggested that the NK cell were activated via NCR1 following infection. To evaluate the extent of NK cell activation we investigated the expression of CD107a and IFNγ production following bacterial challenge. CD107a expression on NK cell membrane represents the fusion of secretory granules with the plasma membrane and characterize early stages of NK cell activation [29]. Three hours following inoculation of mice enhancement of CD107a expression on NCR1-expressing NK cells was higher than that found on NCR1-deficient NK cells (Fig. 3A). In accordance with the CD107a levels, 3h following infection the IFNγ transcript level in the lungs was significantly higher in *Ncr1* ^+/gfp^ mice compared to *Ncr1* ^gfp/gfp^ infected mice (Fig. 3B). In contrast, 3h following infection, TNFα and IL-6 transcripts in the lungs of *Ncr1* ^gfp/gfp^ mice were significantly higher compared to the transcripts of these cytokines in *Ncr1* ^+/gfp^ mice (Fig. 3C, D). This could be explained by the significantly higher bacterial load in *Ncr1* ^gfp/gfp^ mice 3h after infection (Fig. 2A). Indeed, we did not observe this result for TNFα and IL-6 in the *in vitro* assays (data not shown) since *S. pneumoniae* was removed after 1.5 hrs and antibiotics were added.

A recent study suggested that NKp44 may be involved in the direct recognition of bacteria [31]. To investigate the possibility of direct recognition of the bacterium by NCR1 receptor, we stained *S. pneumoniae* with soluble mNCR1-Ig and mNKG2D-Ig fusion proteins (Fig. 7A). Our results suggest the absence of NCR1 ligands *on S. pneumoniae* and thus preclude NCR1-mediated direct recognition of the bacteria by NK cells. However, these results suggest that the enhanced *S. pneumoniae* clearance may be mediated by innate immune accessory cells. The first immune cells to encounter the bacteria are the lung macrophages and DC [2]. To investigate macrophages and DC involvement in the *S. pneumoniae* clearance at early stages following infection we compared the bacterial load level of *S. pneumoniae* in *Ncr1* ^gfp/gfp^, *Ncr1* ^+/gfp^ mice and in DTR:CD11c transgenic mice in which the lung mononuclear phagocytes including alveolar macrophages were ablated (Fig. 5). Our findings suggests that (i) the presence of macrophages and dendritic cells in the lungs is imperative for early clearance *S. pneumoniae*; (ii) the lack of lung mononuclear phagocytes and the absence of NK-expressed *Ncr1* result in the same bacterial load level. Our findings are supported by Sun *et al*. who demonstrated that the resident alveolar macrophages bind *S. pneumoniae* within 4 hours and their depletion with liposomal clodronate led to enhanced bacterial outgrowth in both lung tissues and alveoli [34]. We then searched for the existence of NCR1 receptor ligands on BMMQ and BMDC. Using the mNCR-Ig fusion protein we have found that BMMQ and BMDC express ligands for NCR1 receptor as well as ligands for mNKG2D receptor (Fig. 6A–D). Next we investigated whether the cross talk between NK cells and *S. pneumoniae* infected macrophages and DC is mediated through NCR1 by assessing the extent of IFNγ production. Supernatants of *S. pneumoniae* infected BMMQ and BMDC co-cultured with NK cells showed enhanced IFNγ levels in an NK-NCR1-dependent manner (Fig. 6E–F). We further demonstrated that upon challenge with *S. pneumoniae,* BMDC up-regulate their co stimulatory molecules (data not shown). This up-regulation was previously demonstrated to be useful for their interaction with NK cells which then improves the immune response against bacterial infections [35], [36]. Together these results imply the importance of the cross talk between DC, macrophages and NK cells following infection with *S. pneumoniae in vitro*. According to Ferlazzo *et al*., the biological relevance of NK cell activation mediated by DCs during bacterial infections resides mainly in the secretion of IFNγ [36]. The higher secretion of IFNγ found in our study *in vitro* and *in vivo* in the *Ncr1* ^+/+^ mice may result also from the improved NK-DC/macrophage cross talk which in turn augments bacterial clearance by the DC/macrophages. Our results suggest that NK-expressed NCR1 is mediating a direct cross talk between NK cells and macrophages/DC which is contributing to *S. pneumoniae* clearance by macrophages and DC. Reciprocal activating interaction between NK cells and DC was already reported for several bacterial pathogens [35]–[37]. Yet, our results extend it to NK-macrophages interaction for *S. pneumoniae* infection and point to the involvement of NK-expressed NCR1 in this cross talk. To further assess the role of NK cells and NCR1 in resistance to *S. pneumoniae* infection, NK cells were depleted from *Ncr1* ^gfp/gfp^ and *Ncr1* ^+/+^ mice using anti-asialo GM1 and then challenged with *S. pneumoniae* (Fig. 4). In both strains, depletion of NK cells prior to infection resulted in a significant increased bacterial load 3 h following inoculation in comparison to the mock-treated mice. Surprisingly, the bacterial load level in the lungs of NK-depleted *Ncr1* ^gfp/gfp^ mice was significantly higher than in the NK-depleted *Ncr1* ^+/+^ mice. These results suggest that in addition to NK cells contribution to the early clearance of *S. pneumoniae* in the first 3 h, *Ncr1* ^+/+^ mice are better conditioned to clear *S. pneumoniae*. Indeed, alveolar macrophages from NCR1-positive mice were more potent than alveolar macrophages from NCR1-deficient mice prior to any *S. pneumoniae* infection (Fig. 8). This conditioning may occur in the lung prior to infection and could indicate NCR1-mediated pathogen-independent priming of lung macrophages by NK cells. Alternatively, it could be the result of NCR1-mediated cross talk between NK cells and lung macrophages in response to commensal pathogens. Recently published studies [38], [39] described that activation of NK cells prior to infection resulted in macrophage priming, which improved their microbial clearance abilities. We therefore investigated the expression of NCR1 ligands on macrophages and DCs. The results show, for the first time, a unique macrophage sub-population, located in the BM and in the lungs, which is characterized by a high expression of the NCR1 ligand (Fig. 7, B–F). This population was significantly higher in macrophages from *Ncr1* ^+/+^ and *Ncr1* ^+/gfp^ mice compared to *Ncr1* ^gfp/gfp^ mice. We did not observe this correlation for DC (Fig. 7G). This NCR1-ligand^high^ macrophage sub-population was responsible for the better phagocytic activity of the NCR1-exprsseing mice as it manifested efficient phagocytosis compared to the NCR1-ligand^none^ macrophages (Fig. 8). Therefore, this NCR1-ligand^high^ macrophage sub-population could be involved in the conditioning/priming and/or activation of the macrophages by NCR1-expressing NK cells in a cell-to-cell contact interaction prior to infection and during the early phase of infection.

To summarize, we showed the involvement of NK cells and NK-expressed NCR1 in the early clearance of *S. pneumoniae* and the contribution of NCR1 to the cross talk of NK and macrophages/DC. The plausible phenomenon of NK-NCR1-mediated pre-conditioning of lung phagocytes and the NCR1-dependent sub-population of NCR1-ligand^high^ macrophages should be further explored.
